# Femoral Anteversion in Total Hip Arthroplasty: Retrospective Comparison of Short- and Straight-Stem Models Using CT Scans

**DOI:** 10.3390/jcm12062391

**Published:** 2023-03-20

**Authors:** Sebastian Martin Klim, Patrick Reinbacher, Maria Anna Smolle, Andrzej Hecker, Michael Maier, Joerg Friesenbichler, Andreas Leithner, Lukas Leitner, Alexander Draschl, Jan Lewis, Kevin Brunnader, Werner Maurer-Ertl

**Affiliations:** 1 Department of Orthopaedics & Traumatology, Medical University of Graz, Augenbruggerplatz 5, 8036 Graz, Austria; 2 Division of Plastic, Aesthetic and Reconstructive Surgery, Department of Surgery, Medical University of Graz, Auenbruggerplatz 5, 8036 Graz, Austria

**Keywords:** CT-scan, femoral anteversion, hip geometry reconstruction, short-stem, total hip arthroplasty

## Abstract

Data on reconstruction of the femoral anteversion (FA) and the center of rotation after total hip arthroplasty (THA) are rare. We aimed to answer whether a short-stem fixation enables improved anatomical reconstruction of the FA compared to a straight-stem. Methods: One hundred and thirty patients who underwent short- (n = 89, group A, prospective) or straight-stem THA (n = 41, group B, retrospective) were included. CT scans of the hip, knee, and ankle were performed pre- and postoperatively in group A and in group B during the last follow-up. Femoral torsion was determined using three-dimensional models. Results: The mean preoperative FA was 22.4° ± 11.0°, and the mean postoperative FA was 23.4° ± 10.1°. The relative difference was −0.8° ± 8°, and the absolute difference was 6.4° ± 4.9°. Gender analysis revealed significant differences in preoperative FA between female (f) and male (m) patients (28.1° ± 11.2° (f) vs. 18.4° ± 8.3° (m); *p* > 0.001) as well as in postoperative FA (26.7° ± 23.5° (f) vs. 21.0° ± 9.7° (m); *p* < 0.007) in group A. Postoperative FA was higher in group A (mean 6.8°; 23.9° ± 10.1° (f) vs. 16.6° ± 8.6° (m); *p* < 0.001). Conclusions: The study’s findings suggest that short-stem THA leads to improved anatomical FA reconstruction; however, a substantial postoperative gender-related FA difference was detectable, which may warrant consideration by surgeons when determining the final stem anteversion. It should be noted that the impact of the postoperative gender-related FA difference on clinical outcomes is not entirely clear, and further research is warranted to elucidate this relationship.

## 1. Introduction

Total hip arthroplasty (THA) is considered to be the most successful orthopedic surgery of the 20th century, and it significantly impacts healthcare economics [1]. To achieve optimal results, the femoral stem fixation should satisfy several requirements, including ease of handling during surgery, preservation of bone stock and soft tissue, and stable long-term fixation [2,3,4]. Not only should the surface texture, geometric configuration, and choice of implant material be carefully considered, but the appropriate operative approach is also necessary in order to achieve the best possible results [5]. Furthermore, reconstructing the individual’s hip geometry is crucial in order to achieve optimal force distribution and range of motion while minimizing the risk of aseptic loosening, component wear, and dislocation [6,7,8]. This is emphasized as an important consideration in the literature [9,10,11]. The accurate performance of a THA depends on several criteria that are not considered in a comprehensive manner in conventional THA planning. As Habor et al. suggested in their work, a patient-specific morphofunctional planning of the target zone for implants could provide a solution [12]. Another approach to reach as accurate of a result as possible in THA could be the implementation of image-based robotic assistance throughout the implantation process. Surgeons could make use of haptically controlled robotic arms to achieve more precise results in THA [13].

One of the critical goals of hip anatomy reconstruction during THA is to restore the femoral torsion (ante- or retroversion of the femoral neck) and, consecutively, the center of rotation of the femoral head [14,15]. Improper alignment of component torsion, whether excessive ante- or retrotorsion, can result in impingement and hip instability, leading to complications such as dislocation, component wear, and limited range of motion [16,17,18]. Moreover, failing to restore the angle of femoral anteversion (FA) to 15–20° or to adapt it to the native femoral anteversion or cup anteversion [19,20,21] is associated with a higher risk of dislocation, edge loading, squeaking, hip instability, and limited range of motion [14,17,22]. Therefore, correct alignment of the FA is crucial when trying to achieve an impingement-free range of motion and prevent common complications associated with THA. This can be accomplished by a patient-specific preoperative 3D simulation of range of motion (ROM), flexion, and internal rotation (IR) angle to establish the correct implantation zone of the implant components [23]. Another approach to achieve even better results with THA that are more similar to the naturally occurring physiology would be a patient-specific instrument system that can be individually manufactured for each configuration of the femur and acetabulum [24]. Furthermore, the correct choice of material and implantation site may be crucial when considering a patient’s body mass index (BMI) or the implementation of intraoperational fluoroscopy for the success of THA [25,26].

Moreover, it is known that stem design plays a critical role in determining the stem’s final position and, as a result, its anteversion [27]. Over the course of time, various stem and cup designs were developed to achieve precise hip joint reconstruction and to extend the lifetime of hip implants [27,28]. However, due to the continuous development of new implant designs for cementless short-stem THA as well as its increased use and related research, this method is more prominent [29,30,31]. Although short-stem designs gained popularity due to their ability to preserve proximal femoral bone stock and provide more natural loading in the proximal femur than straight-stems [32,33,34,35,36], only a limited number of studies have directly compared the two designs in terms of FA reconstruction [27,37,38], with short-stems showing higher FA restoration accuracy than straight-stems [38]. However, more data is needed to verify the superiority of short-stem designs in FA reconstruction and their clinical relevance, considering that various stem designs may affect the parameters of hip geometry reconstruction differently. Furthermore, it is important to note that, to date, no study has investigated the influence of the novel, metaphyseal-anchoring, calcar-guided, neck-sparing short-stem designs (ANA.NOVA^®^ Alpha Schaft^®^ Proxy, ImplanTec GmbH, Moedling, Austria) on FA restoration compared to a conventional straight-stem.

Therefore, our study aimed to (1) investigate if a novel calcar-guided short-stem design enables an improved anatomical reconstruction of FA compared to a straight-stem design and (2) whether this effect results in a difference in postoperative clinical and patient-reported outcome measures. We hypothesized that the short-stem designs would restore FA more accurately and result in better outcome measures.

## 2. Materials and Methods

The current study, categorized as level III evidence, involved a retrospective comparative analysis of 130 prospectively included unilateral THA patients who received either a short- or straight-stem design between 2005 and 2017 at a single institution. The anterolateral approach to the hip [39], as recently mentioned by Reinbacher et al. [40], was performed as the standard procedure in both groups, and all patients were operated on for primary hip osteoarthritis. Our research group previously described the characteristics of both stem designs [14]. The current study was approved by the Ethics Committee of the Medical University of Graz, Austria (protocol code 28-152 ex 15/16).

In group A, 89 patients underwent unilateral short-stem THA performed by a single surgeon at a single institution between 2016 to 2017. In this group, all patients were implanted with a cementless short-stem design (ANA.NOVA^®^ Alpha Schaft^®^ Proxy, ImplanTec GmbH, Moedling, Austria) that features epi-metaphyseal fixation combined with a press-fit cup (ANA.NOVA^®^ Alpha Pfanne, ImplanTec GmbH, Moedling, Austria). The short-stem is available in twelve sizes, ranging from zero to eleven, and is designed for neck-shaft angles ranging from 125° to 140°. No alternative designs were provided regarding offset and collar. Pre- and postoperatively, each patient underwent low-dose rotational computed tomography (CT) imaging of the hip, knee, and ankle.

In group B, 41 THA patients received a collarless, cementless straight-stem design with a meta-diaphyseal fixation combined with a press-fit cup (Corail^®^ stem and Pinnacle^®^ cup DePuy Synthes, West Chester, PA, USA) at our department between 2005 and 2012. These patients were selected at random from our follow-up registry to serve as a comparison group, with the condition that postoperative CT scans would be available. The straight-stem design used in this group is available in 13 different sizes with two collar options (with and without) and neck-shaft angle variations (standard or high offset 135°, coxa vara 125°). Complete postoperative rotational CT scans of the hip, knee, and ankle were obtained in this group, but no preoperative CT scans were available. The study excluded pregnant patients, patients under custodianship, or patients with a confirmed periprosthetic joint infection from both groups (A and B).

**Figure 1:**
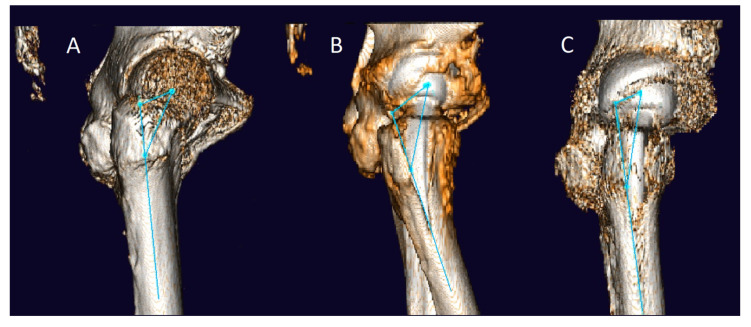
Preoperative and postoperative CT measurement method. 3D measurements obtained with mediCAD hip 3D software, showing the proximal femoral long axis defined by two points and the femoral anteversion angle formed between a perpendicular line through the proximal femoral long axis and the femoral head, and a line connecting the femoral condyles. (**A**): preoperative condition. (**B**): postoperative condition with short stem. (**C**): postoperative condition with straight stem.

To assess FA, 3D measurements were performed using the Hectec mediCAD hip 3D^®^ software (mediCAD Hectec GmbH, Altdorf, Germany). The CT scan images were converted into three-dimensional digital models during this process. The FA was measured preoperatively (only in group A) and postoperatively (in both groups) using the axial oblique technique of Jarrett et al. [41]. Known for its particularly high intra- and interobserver agreement, this measurement technique uses oblique femoral slices with a slice distance and thickness of 5mm [42]. To measure femoral torsion, the angle between a proximal line (aligned with the femoral neck) and a distal femoral line (which is tangential to the posterior condyles on a single axial image with maximum anterior-posterior expansion) is used (Figure 1). Preoperative planning in terms of determining the optimal implant size and position was performed on standard anterior–posterior x-rays of the hip using the mediCAD^®^ Classic Hip 2D software (Hectec GmbH, Altdorf, Germany) as described in a previously published study [43].

3D measurements with mediCAD hip 3D. The femoral long axis is defined by two points in the proximal femur: (1) inferior border of the lesser trochanter; (2) at a point approximately 6 cm distal in the femoral shaft. The angle between the perpendicular line between the proximal femoral long axis and the femoral head and the line between the formal condyles is the femoral anteversion.

The surgeon aimed for a cup inclination between 30° and 50°, a cup anteversion of 10° to 20°, and a stable press-fit fixation. The femoral neck osteotomy for the two implants was performed at different resection levels according to the manufacturer’s recommendations. This generally resulted in a more distal resection height of the femoral neck in group B and a more proximal, bone-preserving resection height in group A. When broaching the femur, the biggest possible stem size was used to attain secure fixation aiming for 15° of FA. In both groups, all stems were combined with cementless cups and ceramic-on-ceramic bearings. Demographic data (age at the time of surgery, gender, and body mass index (BMI)) were recorded. In addition, Western Ontario and McMaster Universities Osteoarthritis Index (WOMAC) scores as well as Harris Hip Scores (HHS) were obtained in the follow-up examinations one year postoperatively from all included patients [44,45].

To detect significant differences, paired and unpaired *t*-tests were used. The Mann–Whitney U test was performed if parametric distribution was not given. Regression analysis was used to detect differences in continuous variables. An alpha level <0.05 was considered significant. All evaluations were done with the statistical program Stata/MP 13.0 (StataCorp, College Station, TX, USA).

## 3. Results

The demographic data and results are shown in Table 1. The mean age at the time of surgery was 60.4 ± 7.5 years in group A and 63.9 ± 10.3 in group B (*p* = 0.03). The mean BMI was 28.5 ± 4.8 (group A) and 28.2 ± 4.5 (group B, *p* > 0.05). In group A, the preoperative FA was 22.4° ± 11.0°, and the postoperative FA was 23.4° ± 10.1°. The relative difference was −0.8° ± 8°, and the absolute difference was 6.4° ± 4.9°. There was no difference regarding the absolute FA change angle from pre- to postoperative phases in group A (7.6° ± 5.7° (f) vs. 5.6° ± 4.0° (m); *p* = 0.057). Furthermore, there was no significant correlation between the change of angle in pre- to postoperative FA and patient age (*p* = 0.657) or body mass index (*p* = 0.307) in this group. When comparing both groups, the postoperative FA was found to be higher in group A than in group B (mean 6.8°; 23.9° ± 10.1° (A) vs. 16.6° ± 8.6° (B); *p* < 0.001; Figure 1).

Fifty-one (57.3%) men were included in group A, and twenty-five (60.9%) were included in group B (*p* = 0.693). Gender analysis in group A revealed significant differences between women (f) and men (m) in the preoperative FA phase (28.1° ± 11.2° (f) vs. 18.4° ± 8.3° (m); *p* > 0.001) as well as in the postoperative FA phase (26.7° ± 23.5° (f) vs. 21.0° ± 9.7° (m); *p* < 0.007). No such differences were found in group B when comparing the postoperative FA (17.9° ± 9.9° (f) vs. 15.7° ± 7.6° (m); *p* = 0.425), as depicted in Table 1.

The HHS in the one-year follow-up was 95.8 ± 8 for group A and 93.5 ± 10 for group B, showing no statistically significant difference between them (*p* = 0.16). The regression analysis for group A showed no significant correlation between the absolute change of angle in the femoral anteversion from the pre- to postoperative phases and the HHS (*p* = 0.50). Furthermore, the regression analysis showed a significantly lower HHS for female patients (*p* > 0.01) and a higher HHS for patients with a higher postoperative FA (*p* = 0.03). The WOMAC score in the one-year follow-up was 10.5 for group A and 9.7 for group B and showed no significant difference between them (*p* = 0.75).

## 4. Discussion

In this retrospective comparative analysis, we examined whether a calcar-guided short-stem design or a conventional straight-stem design would result in better FA reconstruction after THA. We also analyzed whether both stem designs differ regarding patient-reported outcome measures. The results confirmed our hypothesis that the FA was better restored with the short-stem design, but it did not confirm superior clinical outcomes (HHS) or patient-reported outcome measures (WOMAC).

The main finding of this study was the significant difference in anatomical FA reconstruction after THA when comparing a calcar-guided short-stem design with a straight-stem design. As only 17 preoperative CT scans of the contralateral native hip were available in group B, we cannot generalize these results, but in that small number, the preoperative FA was non-significantly different from that of group A (22.4° ± 11 (A) vs. 22.6° ± 8 (B); *p* > 0.05). In a similar preoperative FA situation, the implantation of the calcar-guided short-stem design led to superior FA reconstruction accuracy. This result is in accordance with the findings of Sariali and Pascal Mousselard [38], who compared an anatomic, cementless, and proximally hydroxyapatite (HA)-coated short-stem design (SPS Evolution, Symbios SA, Yverdon-les Bains, Switzerland) to a generic straight-stem design (HARMONY, Symbios SA, Yverdon-les Bains, Switzerland) similar to the straight-stem design investigated in our study. Therefore, our findings provide evidence supporting the effectiveness of short-stem designs in achieving superior FA reconstruction compared to straight-stems while also yielding comparable clinical and patient-reported outcome measures. The results of our study emphasize the effectiveness of the short-stem design, particularly the calcar-guided design that was included in our analysis. The short-stem design aims to reconstruct the hip anatomy more accurately by following the femoral neck’s calcar, thereby improving proximal fit while maintaining femoral anteversion [38,46,47].

The difference in higher FA when using a short-stem design became even more distinct in the gender-based subgroup analysis, as female patients had a significantly higher FA before surgery (28.1° ± 11.2° (f) vs. 18.4° ± 8.3° (m), group A) than their male counterparts. This observation in men was previously reported by Nakahara et al. (25.2  ±  9.8° (f) vs. 20.3  ±  9.9° (m)) and others [48,49,50]. Similarly, a gender-related difference in postoperative FA was only significant in group A. This also lines up with the significantly lower overall postoperative FA in group B. Therefore, optimal reconstruction of the FA seems to be particularly important in women, as the FA may play a role in the significantly higher dislocation rates of women compared to male patients (4:1) after THA [51,52,53,54].

Furthermore, Yoon et al. [55] reported that using a short-stem design increased anterior femoral tilt in the sagittal plane compared to a straight-stem design, which is associated with a higher risk of posterior impingement and anterior dislocation. On the other hand, Fischer et al. [27] reported a higher frequency of postoperative retrotorsion with a collarless straight-stem design compared with a short-stem design, which bears an increased risk of posterior dislocation. Based on current knowledge, these findings suggest that surgeons may need to aim for different femoral anteversion angles for the implanted short-stem design in women and men, indicating the importance of gender-specific considerations. However, dislocation rates were not recorded in the current study; thus, no definitive conclusions can be drawn on this matter. Future studies are needed to investigate this hypothesis further.

However, Faizan et al. [56] discovered a bimodal distribution of anteversion angles in implanted short-stem designs and a difference between pre- and post-virtual implantation anteversion angles while investigating the ABG II monolithic stem system (Stryker Orthopaedics, Mahwah, NJ, USA). Their findings suggest that THA patients may benefit from being divided into two groups, one requiring an anteverted stem and the other requiring less or no anteversion in the stem, to achieve the correct version during FA reconstruction. As the study conducted by Faizan et al. [56] does not offer sufficient information to confirm if the bimodal distribution is related to gender, we cannot determine whether it supports our hypothesis that women may have different postoperative FA requirements than men and could benefit from a stem design with little or no anteversion. Nevertheless, we did observe a significantly higher postoperative FA among women in group A, suggesting that there may be a basis for our hypothesis.

The clinical and patient-reported outcome measures evaluated in this study did not reveal any significant differences between the two groups one year after surgery. Although the HHS suggested better results in group A (*p* = 0.16) and regression analysis revealed significantly better HHS for patients with higher postoperative FA, the WOMAC score did not favor either group (*p* = 0.75). Previous studies also reported this by comparing the postoperative HHS and WOMAC scores of short- and straight-stem THA designs [57,58]. This was to be expected, as the biggest hazards of insufficiently reconstructed hip geometry mainly develop after a longer follow-up period and are usually detected radiographically (aseptic loosening, dislocation, and wear).

Overall, the results of our investigation suggest a more anatomical reconstruction of the FA after THA when using a short-stem design. However, long-term results are needed to investigate whether this improved alignment of the FA significantly impacts the rate of serious adverse events (aseptic loosening, component wear, and dislocation), patient satisfaction, and quality of life. The significant value of this work should be emphasized, as it represents one of the largest series in which the postoperative hip geometries of a short-stem and a straight-stem design were directly compared using state-of-the-art CT scans and 3D measurement techniques. Furthermore, this is the first study to provide data on FA reconstruction using a cementless short-stem design (ANA.NOVA^®^ Alpha Schaft^®^ Proxy, ImplanTec GmbH, Moedling, Austria) in combination with a press-fit cup (ANA.NOVA^®^ Alpha Pfanne, ImplanTec GmbH, Moedling, Austria).

Regarding implantation breakdown in patients with a BMI categorized as obese (class I–III), Ammarullah et al. [25] suggested that material, the texture of the surface, and the use of special coatings should be considered. In addition, they said that the implant geometry and the adaption of surgical procedures to prevent the failure of implantations in obese patients should be kept in mind [25]. In terms of materials and surgical techniques, further research is necessary.

In order to improve the accuracy regarding the as-physiological-as-possible placement of the stem in total hip arthroplasty (THA), the use of patient-specific instrumentation with laser guidance to reduce the risk of femoral anteversion should be considered. Ferretti et al. [5] demonstrated that using a positioning system enables the accurate positioning of the stem and cup. When applied, operating times are not significantly prolonged and even improve in correlation with the user’s learning curve [5]. Free et al. [59] were able to determine that radiological markers can be used in the specific case of the increasingly popular direct anterior approach (DAA) to predict implant malpositioning. It can be inferred that by adapting the surgical technique used, possible positioning errors can be avoided. Furthermore, in certain femur configurations, such as coxa profunda (lower femoral neck-shaft angle and higher lateral center-edge angle), a higher probability of implant malpositioning in THA was described [59]. According to Habor et al. [12], another approach to avoid an unphysiological FA outcome could be the implementation of morphofunctional planning for patient-specific THAs. In this approach, a 3D model-based calculation of the target zone of the joint head is specifically used for each individual patient to prevent poor THA outcomes [12].

Mitsutake et al. [23] showed the role that preoperative 3D imaging can have on range of motion (ROM), considering that a simulated ROM, including flexion and internal rotation angles during the preoperative planning process, can reduce the risk of posterior dislocation of the cup and, consequently, reduce the risk of non-anatomical femoral FA [23]. Another approach to increase the accuracy of THA implantation and consequently avoid unphysiological FA outcomes could be the incorporation of image-guided robotic assistance. Foissey et al. [13] demonstrated that haptically controlled robotic arms allow more precise cup implantation in patients in whom a direct anterior approach (DAA) was performed. This technique could also be helpful in the future for the implantation of stems and could be implemented throughout the whole process of THA [13].

Furthermore, intraoperative fluoroscopy was shown to have better outcomes in terms of unphysiological versions and inclinations in THA than in patients who did not undergo intraoperative fluoroscopy. Consequently, it was demonstrated that intraoperative fluoroscopy imaging enables proper abduction and version of the acetabular cup position. As a result, the desired positioning of THA components could be achieved without a significant extension of the operation time [26].

Zhang et al. [24] demonstrated that the use of a patient-specific instrumentation system promises advantages in the accuracy of implantation compared to freehand THA. Preoperatively acquired CT images are once again used, as in other procedures, and 3D models are molded afterward. These models can be applied as a guide on the femoral neck or acetabulum to ensure accurate osteotomy and, consequently, accurate implantation [24]. Due to the fact that the exact positioning of the stem and the avoidance of unphysiological FA is a matter of multimodal influences, further research in this field will be necessary.

A major limitation of this study is that group B did not undergo any preoperative CT scans of the side of the hip to be operated on. Therefore, an analysis regarding preoperative differences in FA between the two groups could not be performed. Nevertheless, CT scans of the contralateral native hip were available for 17 cases in group B, and the measurements did not show any significant difference in preoperative FA between the two groups (22.4 ± 11° (A) vs. 22.6° ± 8 (B)). Moreover, it is highly improbable that there was a significant preoperative difference in FA because patients received either a short- or straight-stem design based only on the year the surgery was performed. Another limitation is the asymmetric group size of 89 to 41 patients due to the availability of postoperative CT scans in group B bearing the risk of inaccuracies. Furthermore, owing to the short follow-up of clinical and patient-reported outcome measures, the study does not provide data on the long-term effects of different FAs between both groups. Additionally, dislocation rates and rates of ante- or retrotorsion after THA were not recorded; thus, this limits the validity of the superior FA reconstruction outcomes with short-stem designs. Lastly, only one type of short-stem design was analyzed; therefore, the results obtained may not be entirely comparable to other short-stem designs. Hence, the results of this study should be interpreted with caution and in light of its limitations.

## 5. Conclusions

The present study shows that, compared to a predominantly diaphyseal anchoring straight-stem design, a mainly metaphyseal anchoring short-stem design allows for improved anatomical reconstruction of the femoral anteversion in THA. This may be important in the female femoral anatomy for adequate reconstruction of the hip geometry due to their higher variability in femoral antetorsion. This study was able to confirm research from Sariali and Pascal Mousselard [38] regarding the implantation of the calcar-guided short-stem design, which led to superior FA reconstruction accuracy. Regarding materials and surgical techniques for obese THA patients (class I–III), further research is required [25].

## Figures and Tables

**Table 1:** Demographic and clinical data.

		Proxy (n = 89) N; %	Corail (n = 41) N; %	*p*-Value
Gender	Male	51 (57.3)	25 (61.0)	0.693
	Female	38 (42.7)	16 (39.0)	
Hip Type	Coxa vara (CCD < 125°)	47 (52.8)	0 (0.0)	**<0.001**
	Coxa norma (CCD 125–134.9°)	33 (37.1)	0 (0.0)	
	Coxa valga (CCD ≥ 135°)	9 (10.1)	41 (100.0)	
Age at Surgery (in years; mean ± standard deviation)		60.4 ± 4.5	63.9 ± 10.3	**0.030**
BMI (mean ± standard deviation)		28.5 ± 4.8	28.2 ± 4.5	0.603
Preoperative Femoral Anteversion (mean ± standard deviation)		22.4° ± 11.0°	N/A	N/A
Postoperative Femoral Anteversion (mean ± standard deviation)		23.4° ± 10.1°	16.6° ± 1.3°	**<0.001**
HHS Score after 1 year (mean ± standard deviation)		95.8 ± 8.0	93.5 ± 10.1	0.159
WOMAC Score after 1 year (mean ± standard deviation)		10.5 ± 13.6	9.7 ± 14.4	0.758

Significant *p*-values are in bold text.

## Data Availability

The datasets generated and/or analyzed during the current study are available from the corresponding author upon reasonable request.
